# Prediction of intracellular metabolic states from extracellular metabolomic data

**DOI:** 10.1007/s11306-014-0721-3

**Published:** 2014-08-14

**Authors:** Maike K. Aurich, Giuseppe Paglia, Óttar Rolfsson, Sigrún Hrafnsdóttir, Manuela Magnúsdóttir, Magdalena M. Stefaniak, Bernhard Ø. Palsson, Ronan M. T. Fleming, Ines Thiele

**Affiliations:** 1 Center for Systems Biology, University of Iceland, Reykjavik, Iceland; 2 School of Health Science, Faculty of Food Science and Nutrition, University of Iceland, Reykjavik, Iceland; 3 Luxembourg Centre for Systems Biomedicine, University of Luxembourg, Campus Belval, Esch-Sur-Alzette, Luxembourg; 4 Department of Bioengineering, University of California San Diego, La Jolla, CA USA

**Keywords:** Constraint-based modeling, Metabolomics, Multi-omics, Metabolic network, Transcriptomics

## Abstract

Metabolic models can provide a mechanistic framework to analyze information-rich omics data sets, and are increasingly being used to investigate metabolic alternations in human diseases. An expression of the altered metabolic pathway utilization is the selection of metabolites consumed and released by cells. However, methods for the inference of intracellular metabolic states from extracellular measurements in the context of metabolic models remain underdeveloped compared to methods for other omics data. Herein, we describe a workflow for such an integrative analysis emphasizing on extracellular metabolomics data. We demonstrate, using the lymphoblastic leukemia cell lines Molt-4 and CCRF-CEM, how our methods can reveal differences in cell metabolism. Our models explain metabolite uptake and secretion by predicting a more glycolytic phenotype for the CCRF-CEM model and a more oxidative phenotype for the Molt-4 model, which was supported by our experimental data. Gene expression analysis revealed altered expression of gene products at key regulatory steps in those central metabolic pathways, and literature query emphasized the role of these genes in cancer metabolism. Moreover, in silico gene knock-outs identified unique control points for each cell line model, e.g., phosphoglycerate dehydrogenase for the Molt-4 model. Thus, our workflow is well-suited to the characterization of cellular metabolic traits based on extracellular metabolomic data, and it allows the integration of multiple omics data sets into a cohesive picture based on a defined model context.

**Electronic supplementary material:**

The online version of this article (doi:10.1007/s11306-014-0721-3) contains supplementary material, which is available to authorized users.

## Introduction

Modern high-throughput techniques have increased the pace of biological data generation. Also referred to as the “omics avalanche”, this wealth of data provides great opportunities for metabolic discovery. Omics data sets contain a snapshot of almost the entire repertoire of mRNA, protein, or metabolites at a given time point or under a particular set of experimental conditions. Because of the high complexity of the data sets, computational modeling is essential for their integrative analysis. Currently, such data analysis is a bottleneck in the research process and methods are needed to facilitate the use of these data sets, e.g., through meta-analysis of data available in public databases [e.g., the human protein atlas (Uhlen et al. 2010) or the gene expression omnibus (Barrett et al. 2011)], and to increase the accessibility of valuable information for the biomedical research community.

Constraint-based modeling and analysis (COBRA) is a computational approach that has been successfully used to investigate and engineer microbial metabolism through the prediction of steady-states (Durot et al. 2009). The basis of COBRA is network reconstruction: networks are assembled in a bottom-up fashion based on genomic data and extensive organism-specific information from the literature. Metabolic reconstructions capture information on the known biochemical transformations taking place in a target organism to generate a biochemical, genetic and genomic knowledge base (Reed et al. 2006). Once assembled, a metabolic reconstruction can be converted into a mathematical model (Thiele and Palsson 2010), and model properties can be interrogated using a great variety of methods (Schellenberger et al. 2011). The ability of COBRA models to represent genotype–phenotype and environment–phenotype relationships arises through the imposition of constraints, which limit the system to a subset of possible network states (Lewis et al. 2012). Currently, COBRA models exist for more than 100 organisms, including humans (Duarte et al. 2007; Thiele et al. 2013).

Since the first human metabolic reconstruction was described [Recon 1 (Duarte et al. 2007)], biomedical applications of COBRA have increased (Bordbar and Palsson 2012). One way to contextualize networks is to define their system boundaries according to the metabolic states of the system, e.g., disease or dietary regimes. The consequences of the applied constraints can then be assessed for the entire network (Sahoo and Thiele 2013). Additionally, omics data sets have frequently been used to generate cell-type or condition-specific metabolic models. Models exist for specific cell types, such as enterocytes (Sahoo and Thiele 2013), macrophages (Bordbar et al. 2010), and adipocytes (Mardinoglu et al. 2013), and even multi-cell assemblies that represent the interactions of brain cells (Lewis et al. 2010). All of these cell type specific models, except the enterocyte reconstruction were generated based on omics data sets. Cell-type-specific models have been used to study diverse human disease conditions. For example, an adipocyte model was generated using transcriptomic, proteomic, and metabolomics data. This model was subsequently used to investigate metabolic alternations in adipocytes that would allow for the stratification of obese patients (Mardinoglu et al. 2013). One highly active field within the biomedical applications of COBRA is cancer metabolism (Jerby and Ruppin, 2012). Omics-driven large-scale models have been used to predict drug targets (Folger et al. 2011; Jerby et al. 2012). A cancer model was generated using multiple gene expression data sets and subsequently used to predict synthetic lethal gene pairs as potential drug targets selective for the cancer model, but non-toxic to the global model (Recon 1), a consequence of the reduced redundancy in the cancer specific model (Folger et al. 2011). In a follow up study, lethal synergy between FH and enzymes of the heme metabolic pathway were experimentally validated and resolved the mechanism by which FH deficient cells, e.g., in renal-cell cancer cells survive a non-functional TCA cycle (Frezza et al. 2011).

Contextualized models, which contain only the subset of reactions active in a particular tissue (or cell-) type, can be generated in different ways (Becker and Palsson, 2008; Jerby et al. 2010). However, the existing algorithms mainly consider gene expression and proteomic data to define the reaction sets that comprise the contextualized metabolic models. These subset of reactions are usually defined based on the expression or absence of expression of the genes or proteins (present and absent calls), or inferred from expression values or differential gene expression. Comprehensive reviews of the methods are available (Blazier and Papin, 2012; Hyduke et al. 2013). Only the compilation of a large set of omics data sets can result in a tissue (or cell-type) specific metabolic model, whereas the representation of one particular experimental condition is achieved through the integration of omics data set generated from one experiment only (condition-specific cell line model). Recently, metabolomic data sets have become more comprehensive and using these data sets allow direct determination of the metabolic network components (the metabolites). Additionally, metabolomics has proven to be stable, relatively inexpensive, and highly reproducible (Antonucci et al. 2012). These factors make metabolomic data sets particularly valuable for interrogation of metabolic phenotypes. Thus, the integration of these data sets is now an active field of research (Li et al. 2013; Mo et al. 2009; Paglia et al. 2012b; Schmidt et al. 2013). Generally, metabolomic data can be incorporated into metabolic networks as qualitative, quantitative, and thermodynamic constraints (Fleming et al. 2009; Mo et al. 2009). Mo et al. used metabolites detected in the spent medium of yeast cells to determine intracellular flux states through a sampling analysis (Mo et al. 2009), which allowed unbiased interrogation of the possible network states (Schellenberger and Palsson 2009) and prediction of internal pathway use. Such analyses have also been used to reveal the effects of enzymopathies on red blood cells (Price et al. 2004), to study effects of diet on diabetes (Thiele et al. 2005) and to define macrophage metabolic states (Bordbar et al. 2010). This type of analysis is available as a function in the COBRA toolbox (Schellenberger et al. 2011).

**Fig. 1:**
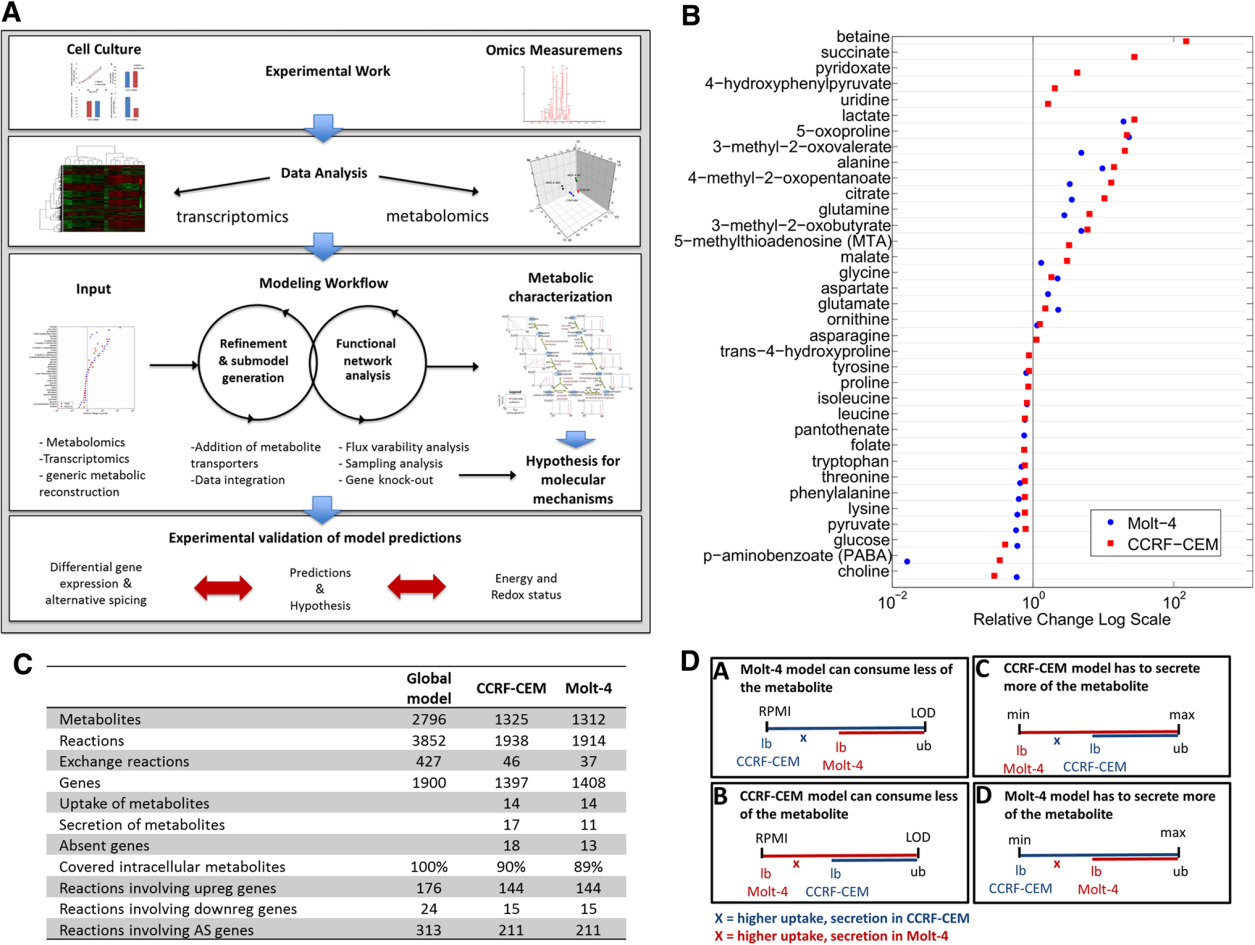
**A** Combined experimental and computational pipeline to study human metabolism. Experimental work and omics data analysis steps precede computational modeling. Model predictions are validated based on targeted experimental data. Metabolomic and transcriptomic data are used for model refinement and submodel extraction. Functional analysis methods are used to characterize the metabolism of the cell-line models and compare it to additional experimental data. The validated models are subsequently used for the prediction of drug targets. **B** Uptake and secretion pattern of model metabolites. All metabolite uptakes and secretions that were mapped during model generation are shown. Metabolite uptakes are depicted on the *left*, and secreted metabolites are shown on the *right*. A number of metabolite exchanges mapped to the model were unique to one cell line. Differences between cell lines were used to set quantitative constraints for the sampling analysis. **C** Statistics about the cell line-specific network generation. **D** Quantitative constraints. For the sampling analysis, an additional set of constraints was imposed on the cell line specific models, emphasizing the differences in metabolite uptake and secretion between cell lines. Higher uptake of a metabolite was allowed in the model of the cell line that consumed more of the metabolite in vitro, whereas the supply was restricted for the model with lower in vitro uptake. This was done by establishing the same ratio between the models bounds as detected in vitro. *X* denotes the factor (slope ratio) that distinguishes the bounds, and which was individual for each metabolite. (a) The uptake of a metabolite could be x times higher in CCRF-CEM cells, (b) the metabolite uptake could be x times higher in Molt-4, (c) metabolite secretion could be x times higher in CCRF-CEM, or (d) metabolite secretion could be x times higher in Molt-4 cells. *LOD* limit of detection. The consequence of the adjustment was, in case of uptake, that one model was constrained to a lower metabolite uptake (A, B), and the difference depended on the ratio detected in vitro. In case of secretion, one model had to secrete more of the metabolite, and again the difference depended on the experimental difference detected between the cell lines

In this study, we established a workflow for the generation and analysis of condition-specific metabolic cell line models that can facilitate the interpretation of metabolomic data. Our modeling yields meaningful predictions regarding metabolic differences between two lymphoblastic leukemia cell lines (Fig. 1A).

## Results

We set up a pipeline that could be used to infer intracellular metabolic states from semi-quantitative data regarding metabolites exchanged between cells and their environment. Our pipeline combined the following four steps: data acquisition, data analysis, metabolic modeling and experimental validation of the model predictions (Fig. 1A). We demonstrated the pipeline and the predictive potential to predict metabolic alternations in diseases such as cancer based on two lymphoblastic leukemia cell lines. The resulting Molt-4 and CCRF-CEM condition-specific cell line models were able to explain metabolite uptake and secretion by predicting the distinct utilization of central metabolic pathways by the two cell lines. Whereas the CCRF-CEM model resembled more a glycolytic, commonly referred to as ‘Warburg’ phenotype, suggested our predictions a more respiratory phenotype for the Molt-4 model. We found these predictions to be in agreement with measured gene expression differences at key regulatory steps in the central metabolic pathways, and they were also consistent with additional experimental data regarding the energy and redox states of the cells. After a brief discussion of the data generation and analysis steps, the results derived from model generation and analysis will be described in detail.

### Pipeline for generation of condition-specific metabolic cell line models

#### Generation of experimental data

We monitored the growth and viability of lymphoblastic leukemia cell lines in serum-free medium (File S2, Fig. S1). Multiple omics data sets were derived from these cells. Extracellular metabolomics (exo-metabolomic) data, comprising measurements of the metabolites in the spent medium of the cell cultures (Paglia et al. 2012a), were collected along with transcriptomic data, and these data sets were used to construct the models.

#### Analysis of experimental data

Data analysis included defining the sets of metabolites that were taken up or secreted (qualitatively for the generation of the models), and it included determining the quantitative differences in uptake and secretion between cell lines (Fig. 1B). These differences were later subjected to model constraints. The final sets of metabolite exchanges that were used for model generation comprised the uptake and secretion of 14 and 10 metabolites by both models, unique secretion of 7 and unique uptake of 4 metabolites by the CCRF-CEM model, and secretion of 1 and uptake of 1 unique metabolite in Molt-4 cells (Fig. 1B). Additionally, sets of genes treated as expressed and unexpressed (absent and present calls), and groups of differentially expressed genes (DEGs) and alternatively spliced genes (AS) were predicted by comparing expression in CCRF-CEM and Molt-4 cells (see “Materials and methods” section and File S2 in supplementary information for more detail).

#### Generation of condition-specific cell line models

Model generation involves three steps: refinement of the global model, data mapping and submodel extraction. We added transport and exchange reactions for metabolites that could not be transported between the extracellular space and the cytosol (see “Materials and methods” section). Nutrient supply (for metabolite uptake) was restricted to the RPMI medium composition (File S1, Table S1).

First, the detected metabolite uptakes and secretions for each cell line were mapped separately to the model. The model was thereby constrained to represent a minimal set of metabolite exchange reactions required to support all of the observed metabolite uptakes and secretions and to explain the experimentally observed growth rates of the cells (Fig. 1B, File S1, Tables S2–S3). The result was a vast reduction of the number of possible metabolite uptakes and secretions in the two preliminary models (Fig. 1C), which placed major emphasis on the experimentally observed metabolite uptake and secretion profiles.

In addition to the (qualitative) exo-metabolomic constraints, genomic data were mapped to the preliminary models (File S1, Table S4). In general, the mapping of transcriptomic data, which meant the deletion of all reactions associated with the set of absent genes, and which was performed after the integration of the exo-metabolomic data, did not prevent that either model could represent the detected metabolite uptake, metabolite secretion, or biomass production. Curation beyond the initial definition of the minimal sets of mandatory exchanges was therefore not necessary.

Subsequently, the condition-specific CCRF-CEM and Molt-4 models were extracted through network pruning. Model reactions unable to support flux were identified through flux variability analysis (FVA) and removed, leaving the functional reaction sets to compose the final Molt-4 and CCRF-CEM models.

#### Condition-specific models for CCRF-CEM and Molt-4 cells

To determine whether we had obtained two distinct models, we evaluated the reactions, metabolites, and genes of the two models. Both the Molt-4 and CCRF-CEM models contained approximately half of the reactions and metabolites present in the global model (Fig. 1C). They were very similar to each other in terms of their reactions, metabolites, and genes (File S1, Table S5A–C). The Molt-4 model contained seven reactions that were not present in the CCRF-CEM model (Co-A biosynthesis pathway and exchange reactions). In contrast, the CCRF-CEM contained 31 unique reactions (arginine and proline metabolism, vitamin B6 metabolism, fatty acid activation, transport, and exchange reactions). There were 2 and 15 unique metabolites in the Molt-4 and CCRF-CEM models, respectively (File S1, Table S5B). Approximately three quarters of the global model genes remained in the condition-specific cell line models (Fig. 1C). The Molt-4 model contained 15 unique genes, and the CCRF-CEM model had 4 unique genes (File S1, Table S5C). Both models lacked NADH dehydrogenase (complex I of the electron transport chain—ETC), which was determined by the absence of expression of a mandatory subunit (NDUFB3, Entrez gene ID 4709). Rather, the ETC was fueled by FADH2 originating from succinate dehydrogenase and from fatty acid oxidation, which through flavoprotein electron transfer could contribute to the same ubiquinone pool as complex I and complex II (succinate dehydrogenase). Despite their different in vitro growth rates (which differed by 11 %, see File S2, Fig. S1) and differences in exo-metabolomic data (Fig. 1B) and transcriptomic data, the internal networks were largely conserved in the two condition-specific cell line models.

#### Condition-specific cell line models predict distinct metabolic strategies

Despite the overall similarity of the metabolic models, differences in their cellular uptake and secretion patterns suggested distinct metabolic states in the two cell lines (Fig. 1B and see “Materials and methods” section for more detail). To interrogate the metabolic differences, we sampled the solution space of each model using an Artificial Centering Hit-and-Run (ACHR) sampler (Thiele et al. 2005). For this analysis, additional constraints were applied, emphasizing the quantitative differences in commonly uptaken and secreted metabolites. The maximum possible uptake and maximum possible secretion flux rates were reduced according to the measured relative differences between the cell lines (Fig. 1D, see “Materials and methods” section).

We plotted the number of sample points containing a particular flux rate for each reaction. The resulting binned histograms can be understood as representing the probability that a particular reaction can have a certain flux value. A comparison of the sample points obtained for the Molt-4 and CCRF-CEM models revealed a considerable shift in the distributions, suggesting a higher utilization of glycolysis by the CCRF-CEM model (File S2, Fig. S2). This result was further supported by differences in medians calculated from sampling points (File S1, Table S6). The shift persisted throughout all reactions of the pathway and was induced by the higher glucose uptake (35 %) from the extracellular medium in CCRF-CEM cells. The sampling median for glucose uptake was 34 % higher in the CCRF-CEM model than in Molt-4 model (File S2, Fig. S2).

**Fig. 2:**
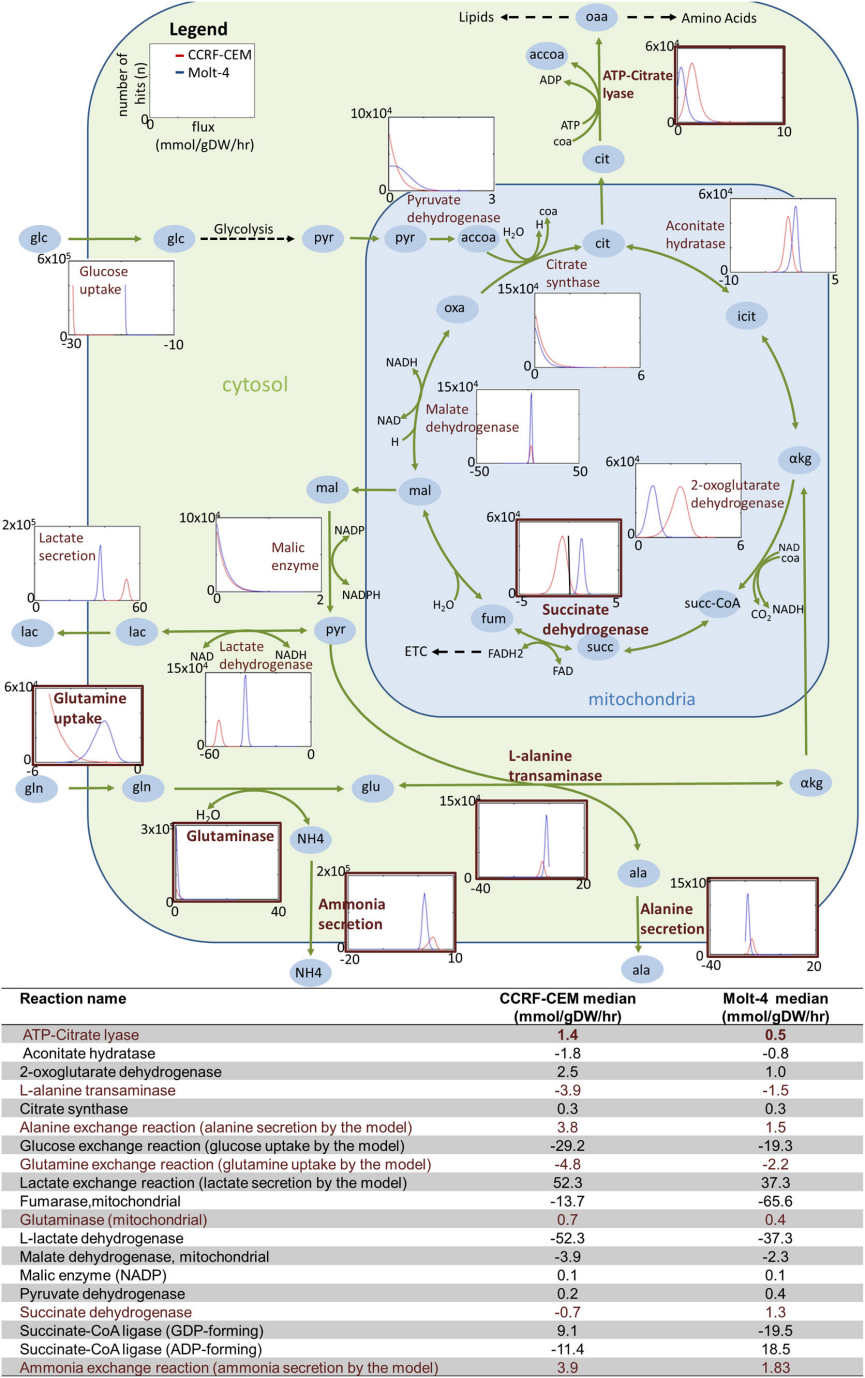
Differences in the use of the TCA cycle by the CCRF-CEM model (*red*) and the Molt-4 model (*blue*). The table provides the median values of the sampling results. *Negative values* in histograms and in the table describe reversible reactions with flux in the reverse direction. There are multiple reversible reactions for the transformation of isocitrate and α-ketoglutarate, malate and fumarate, and succinyl-CoA and succinate. These reactions are unbounded, and therefore histograms are not shown. The details of participating cofactors have been removed. *Atp* ATP, *cit* citrate, *adp* ADP, *pi* phosphate, *oaa* oxaloacetate, *accoa* acetyl-CoA, *coa* coenzyme-A, *icit* isocitrate, α*kg* α-ketoglutarate, *succ*-*coa* succinyl-CoA, *succ* succinate, *fum* fumarate, *mal* malate, *oxa* oxaloacetate, *pyr* pyruvate, *lac* lactate, *ala* alanine, *gln* glutamine, *ETC* electron transport chain

**Fig. 3:**
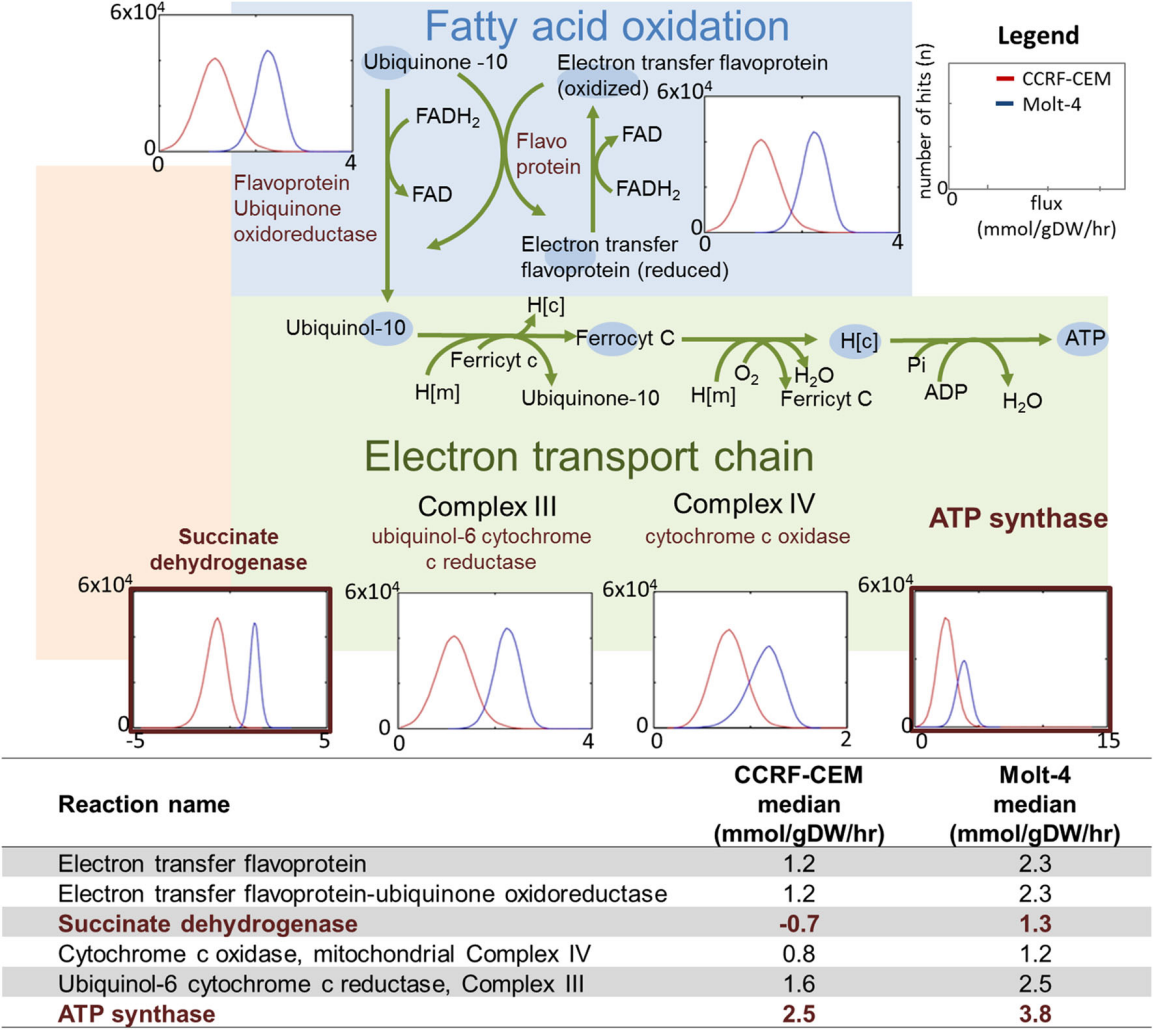
Sampling reveals different utilization of oxidative phosphorylation by the generated models. Different distributions are observed for the CCRF-CEM model (*red*) and the Molt-4 model (*blue*). Molt-4 has higher median flux through ETC reactions II–IV. The table provides the median values of the sampling results. *Negative values* in the histograms and in the table describe reversible reactions with flux in the reverse direction. Both models lack Complex I of the ETC because of constraints arising from the mapping of transcriptomic data. Electron transfer flavoprotein and electron transfer flavoprotein–ubiquinone oxidoreductase both also carry higher flux in the Molt-4 model

The usage of the TCA cycle was also distinct in the two condition-specific cell-line models (Fig. 2). Interestingly, the models used succinate dehydrogenase differently (Figs. 2, 3). The Molt-4 model utilized an associated reaction to generate FADH2, whereas in the CCRF-CEM model, the histogram was shifted in the opposite direction, toward the generation of succinate. Additionally, there was a higher efflux of citrate toward amino acid and lipid metabolism in the CCRF-CEM model (Fig. 2). There was higher flux through anaplerotic and cataplerotic reactions in the CCRF-CEM model than in the Molt-4 model (Fig. 2); these reactions include the efflux of citrate through ATP-citrate lyase, uptake of glutamine, generation of glutamate from glutamine, transamination of pyruvate and glutamate to alanine and to 2-oxoglutarate, secretion of nitrogen, and secretion of alanine. The Molt-4 model showed higher utilization of oxidative phosphorylation (Fig. 3), again supported by elevated median flux through ATP synthase (36 %) and other enzymes, which contributed to higher oxidative metabolism. The sampling analysis therefore revealed different usage of central metabolic pathways by the condition-specific models.

#### Experimental validation of energy and redox status of CCRF-CEM and Molt-4 cells

**Fig. 4:**
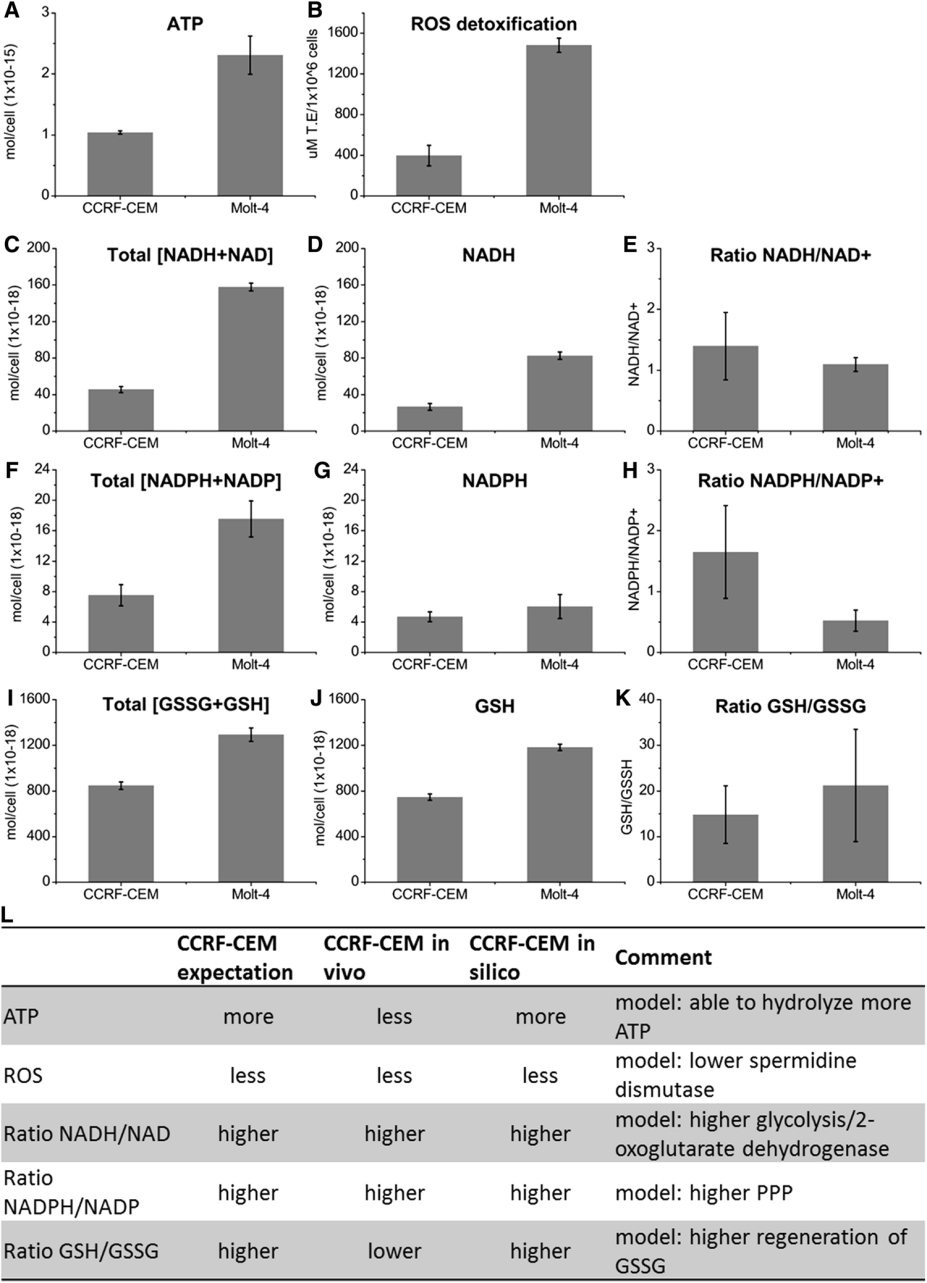
**A–K** Experimentally determined ATP, NADH + NAD, NADPH + NADP, and GSH + GSSG concentrations, and ROS detoxification in the CCRF-CEM and Molt-4 cells. **L** Expectations for cellular energy and redox states. Expectations are based on predicted metabolic differences of the Molt-4 and CCRF-CEM models

Cancer cells have to balance their needs for energy and biosynthetic precursors, and they have to maintain redox homeostasis to proliferate (Cairns et al. 2011). We conducted enzymatic assays of cell lysates to measure levels and/or ratios of ATP, NADPH + NADP, NADH + NAD, and glutathione. These measurements were used to provide support for the in silico predicted metabolic differences (Fig. 4). Additionally, an Oxygen Radical Absorbance Capacity (ORAC) assay was used to evaluate the cellular antioxidant status (Fig. 4B). Total concentrations of NADH + NAD, GSH + GSSG, NADPH + NADP and ATP, were higher in Molt-4 cells (Fig. 4A). The higher ATP concentration in Molt-4 cells could either result from high production rates, or intracellular accumulation connected to high or low reactions fluxes (Fig. 4A). Our simplified view that oxidative Molt-4 produces less ATP and was contradicted by the higher ATP concentrations measured (Fig. 4L). Yet we want to emphasize that concentrations cannot be compared to flux values, since we are modeling at steady-state. NADH/NAD+ ratios for both cell lines were shifted toward NADH (Fig. 4D, E), but the shift toward NADH was more pronounced in CCRF-CEM (Fig. 4E), which matched our expectation based on the higher utilization of glycolysis and 2-oxoglutarate dehydrogenase in the CCRF-CEM model (Fig. 4L).

The mitochondrial membrane has been suggested to be the quantitatively most important physiological source of superoxide in higher organisms (Chance et al. 1979). If the Molt-4 cells were relying more on mitochondrial respiration, we expected them to counteract the increased oxidative stress by using antioxidant systems such as glutathione and NADPH (Fig. 4L). Indeed, Molt-4 cells showed a higher capacity for reactive oxygen species (ROS) detoxification than CCRF-CEM cells (Fig. 4B), which was supported by the higher utilization of oxidative phosphorylation and spermidine dismutase by the Molt-4 model (SPODM, median CCRF-CEM = 0.0010 U, and Molt-4 = 0.0011 U) (Fig. 4L). Reduced glutathione (GSH) is of major importance for the clearance of ROS (Droge 2002). GSH/GSSG ratios were shifted toward GSH in both cell lines (CCRF-CEM = 747:51, Molt-4 = 1182:56), and the shift was more pronounced in Molt-4 cells (Fig. 4K).

Both cell lines had low NADPH/NADP+ ratios (CCRF-CEM 4.7:2.8, Molt-4 6:11.5). However, in Molt-4 cells, the ratio was shifted toward NADP+, whereas CCRF-CEM cells contained higher amounts of NADPH (Fig. 4G, H). This matched our expectation that the glycolytic CCRF-CEM model would produce more NADPH (Fig. 4L) and that it would exhibit higher flux through the oxidative phase of the pentose phosphate pathway (PPP). Taken together, the experimental data agreed well with our expectations based on the predicted phenotypes. We sought additional support for the predicted metabolic differences in the transcriptomic data.

#### Comparison of network utilization and alteration in gene expression

With the assumption that differential expression of particular genes would cause reaction flux changes, we determined how the differences in gene expression (between CCRF-CEM and Molt-4) compared to the flux differences observed in the models. Specifically, we checked whether the reactions associated with genes upregulated (significantly more expressed in CCRF-CEM cells compared to Molt-4 cells) were indeed more utilized by the CCRF-CEM model, and we checked whether downregulated genes were associated with reactions more utilized by the Molt-4 model.

The set of downregulated genes was associated with 15 reactions, and the set of 49 upregulated genes was associated with 113 reactions in the models. Reactions were defined as differently utilized if the difference in flux exceeded 10 % (considering only non-loop reactions). Of the reactions associated with upregulated genes, 72.57 % were more utilized by the CCRF-CEM model, and 2.65 % were more utilized by the Molt-4 model (File S1, Table S7). In contrast, all 15 reactions associated with the 12 downregulated genes were more utilized in the CCRF-CEM model (File S1, Table S8). After this initial analysis, we approached the question from a different angle, asking whether the majority of the reactions associated with each individual gene upregulated in CCRF-CEM were more utilized by the CCRF-CEM model. We found that this was the case for 77.55 % of the upregulated genes. The majority of reactions associated with two (16.67 %) downregulated genes were more utilized by the Molt-4 model. Taken together, our comparisons of the direction of gene expression with the fluxes of the two cancer cell-line models confirmed that reactions associated with upregulated genes in the CCRF-CEM cells were generally more utilized by the CCRF-CEM model.

#### Accumulation of DEGs and AS genes at key metabolic steps

After we confirmed that most reactions associated with upregulated genes were more utilized by the CCRF-CEM model, we checked the locations of DEGs within the network. In this analysis, we paid special attention to the central metabolic pathways that we had found to be distinctively utilized by the two models. Several DEGs and AS events were associated with glycolysis, the ETC, pyruvate metabolism, and the PPP (Table 1).

**Table 1:** DEGs and AS events of central metabolic and cancer-related pathways

DEG associated reactions	Median Molt-4 (mmol/gdw/h)	Median CCRF-CEM (mmol/gdw/h)	Entrez Gene ID	Direction change	Subsystem	AS Entrez Gene ID
ALDD2xm	0.040	0.051	219	Upregulated	Glycolysis/glucon.	
FBA	12.898	18.800	230	Downregulated	Glycolysis/glucon.	
G3PD2 m	0.068	0.191	2820	Upregulated	Glycolysis/glucon.	
PYK	36.115	54.746	5315	Upregulated	Glycolysis/glucon.	
ALDD2x	0.035	0.050	223	Upregulated	Glycolysis/glucon.	8854
ALDD2y	0.039	0.052	223	Upregulated	Glycolysis/glucon.	8854
G6PPer	0.099	0.138	92579	Upregulated	Glycolysis/glucon.	92579
PDHm	0.351	0.162	1737	Upregulated	Glycolysis/glucon.	1737
PFK	13.041	18.995	5213	Downregulated	Glycolysis/glucon.	5211 5213
HEX1	6.217	9.835			Glycolysis/glucon.	3098
PGK	−36.230	−54.935			Glycolysis/glucon.	5230
ALCD21_D	326.100	327.300	284273	Upregulated	Pyruvate met.	284273
ALCD21_L	129.365	128.372	284273	Upregulated	Pyruvate met.	284273
ALCD22_D	291.679	289.357	284273	Upregulated	Pyruvate met.	284273
ALCD22_L	129.260	128.219	284273	Upregulated	Pyruvate met.	284273
LCADi	0.073	0.100	223	Upregulated	Pyruvate met.	8854
LCADi_D	0.072	0.100	223	Upregulated	Pyruvate met.	8854
PCm	0.241	1.300	5091	Downregulated	Pyruvate met.	5091
LALDD	338.276	345.473			Pyruvate met.	9380
ME2 m	0.221	0.178			Pyruvate met.	10873
NADH2_u10 m				Upregulated	OxPhos	
ATPS4 m	3.825	2.455			OxPhos	4905
CYOR_u10 m	2.506	1.563			OxPhos	1537
DRBK	0.146	0.196			PPP	64080
G6PDH2r	0.125	0.109			PPP	2539

Full lists of DEGs and AS are provided in the supplementary material. *Upregulated* significantly more expressed in CCRF-CEM compared to Molt-4 cells

*PPP* pentose phosphate pathway, *OxPhos* oxidative phosphorylation, *Glycolysis/glucon* glycolysis/gluconeogenesis, *Pyruvate met.* pyruvate metabolism

Moreover, in glycolysis, the DEGs and/or AS genes were associated with all three rate-limiting steps, i.e., the steps mediated by hexokinase, pyruvate kinase, and phosphofructokinase. Of these key enzymes, hexokinase 1 (Entrez Gene ID: 3098) was alternatively spliced, and pyruvate kinase (PKM, Entrez gene ID: 5315) was significantly more expressed in the CCRF-CEM cells (Table 1), in agreement with the higher in silico predicted flux. However, in contrast to the observed higher utilization of glycolysis in the CCRF-CEM model, we found that the gene associated with the rate-limiting glycolysis step, phosphofructokinase (Entrez Gene ID: 5213), was significantly upregulated in Molt-4 cells relative to CCRF-CEM cells. This higher expression was detected for only a single isozyme, however. Two of the three genes associated with phosphofructokinase were also subject to alternative splicing (Table 1). In addition to the key enzymes, fructose bisphosphate aldolase (Entrez Gene ID: 230) was also significantly upregulated in Molt-4 cells relative to CCRF-CEM cells, which was in contrast to the predicted higher utilization of glycolysis in the CCRF-CEM model.

Additionally, glucose-6P-dehydrogenase (G6PD), which catalyzes the first reaction and commitment step of the PPP, was an AS gene (Table 1). A second AS gene associated with the PPP reaction of the deoxyribokinase was RBKS (Entrez Gene ID: 64080). This gene is also associated with ribokinase, but ribokinase was removed during model construction because of the lack of ribose uptake or secretion. Single AS genes were associated with different complexes of the ETC (Table 1). Literature query revealed that at least 13 genes associated with alternative splicing events were mentioned previously in connection with both alternative splicing and cancer (File S1, Table S14), and 37 genes were associated with cancer, e.g., upregulated, downregulated at the level of mRNA or protein, or otherwise connected to cancer metabolism and signaling. One general observation was that there was a surprising accumulation of metabolite transporters among the AS.

Overall, the high incidence of differential gene expression events at metabolic control points increases the plausibility of the in silico predictions.

#### Single gene deletion

Analyses of essential genes in metabolic models have been used to predict candidate drug targets for cancer cells (Folger et al. 2011). Here, we conducted an in silico gene deletion study for all model genes to identify a unique set of knock-out (KO) genes for each condition-specific cell line model. The analysis yielded 63 shared lethal KO genes and distinct sets of KO genes for the CCRF-CEM model (11 genes) and the Molt-4 model (3 genes). For three of the unique CCRF-CEM KO genes, the genes were only present in the CCRF-CEM model (File S1, Table S9).

The essential genes for both models were then related to the cell-line-specific differences in metabolite uptake and secretion (Fig. 1B). The CCRF-CEM model needed to generate putrescine from ornithine (ORNDC, Entrez Gene ID: 4953) to subsequently produce 5-methylthioadenosine for secretion (Fig. 1B). *S*-adenosylmethioninamine produced by adenosylmethionine decarboxylase (arginine and proline metabolism, associated with Entrez Gene ID: 262) is a substrate required for generation of 5-methylthioadenosine. Another example of a KO gene connected to an enforced exchange reaction was glutamic-oxaloacetic transaminase 1 (GOT1, Entrez Gene ID: 2805). Without GOT1, the CCRF-CEM model was forced to secrete 4-hydroxyphenylpyruvate (Fig. 1B), the second product of tyrosine transaminase, which is produced only by that enzyme.

One KO gene in the Molt-4 model (Entrez Gene ID: 26227) was associated with phosphoglycerate dehydrogenase (PGDH), which catalyzes the conversion of 3-phospho-d-glycerate to 3-phosphohydroxypyruvate while generating NADH from NAD+. This KO gene is particularly interesting, given the involvement of this reaction in a novel pathway for ATP generation in rapidly proliferating cells (Locasale et al. 2011; Vander Heiden 2011; Vazquez et al. 2011). Reactions associated with unique KO genes were in many cases utilized more by the model, in which the gene KO was lethal, underlining the potential importance of these reactions for the models. Thus, single gene deletion provided unique sets of lethal genes that could be specifically targeted to kill these cells.

## Discussion

In the current study, we explored the possibility of semi-quantitatively integrating metabolomic data with the human genome-scale reconstruction to facilitate analysis. By constructing condition-specific cell line models to provide a structured framework, we derived insights that could not have been obtained from data analysis alone.

We derived condition-specific cell line models for CCRF-CEM and Molt-4 cells that were able to explain the observed exo-metabolomic differences (Fig. 1B). Despite the overall similarities between the models, the analysis revealed distinct usage of central metabolic pathways (Figs. 2, 3, 4), which we validated based on experimental data and differential gene expression. The additional data sufficiently supported metabolic differences in these cell lines, providing confidence in the generated models and the model-based predictions. We used the validated models to predict unique sets of lethal genes to identify weak links in each model. These weak links may represent potential drug targets.

Integrating omics data with the human genome-scale reconstruction provides a structured framework (i.e., pathways) that is based on careful consideration of the available biochemical literature (Thiele and Palsson 2010). This network context can simplify omics data analysis, and it allows even non-biochemical experts to gain fast and comprehensive insights into the metabolic aspects of omics data sets. Compared to transcriptomic data, methods for the integration and analysis of metabolomic data in the context of metabolic models are less well established, although it is an active field of research (Li et al. 2013; Paglia et al. 2012b). In contrast to other studies, our approach emphasizes the representation of experimental conditions rather than the reconstruction of a generic, cell-line-specific network, which would require the combination of data sets from many experimental conditions and extensive manual curation. Rather, our way of model construction allowed us to efficiently assess the metabolic characteristics of cells. Despite the fact, that only a limited number of exchanged metabolites can be measured by available metabolomics platforms and at reasonable time-scale, and that pathways of measured metabolites might still be unknown to date (File S1, Tables S2–S3), our methods have the potential to reveal metabolic characteristics of cells which could be useful for biomedicine and personalized health. The reasons why some cancers respond to certain treatments and not others remain unclear, and choosing a treatment for a specific patient is often difficult (Vander Heiden 2011). One potential application of our approach could be the characterization of cancer phenotypes to explore how cancer cells or other cell types with particular metabolic characteristics respond to drugs.

The generation of our condition-specific cell line models involved only limited manual curation, making this approach a fast way to place metabolomic data into a network context. Model building mainly involves the rigid reduction of metabolite exchanges to match the observed metabolite exchange pattern with as few additional metabolite exchanges as possible. It should be noted that this reduction determines, which pathways can be utilized by the model. Our approach mostly conserved the internal network redundancy. However, a more significant reduction may be achieved using different data. Generally, a trade-off exists between the reduction of the internal network and the increasing number of network gaps that need to be curated by using additional omics data, such as transcriptomics and proteomics. One way to prevent the emergence of network gaps would be to use mapping algorithms that conserve network functionality, such as GIMME (Becker and Palsson 2008). However, several additional methods exist for the integration of transcriptomic data (Blazier and Papin 2012), and which model-building method is best depends on the available data. Interestingly, the lack of a significant contribution of our gene expression data to the reduction of network size suggests that the use of transcriptomic data is not necessary to identify distinct metabolic strategies; rather, the integration of exo-metabolomic data alone may provide sufficient insight. However, sampling of the cell line models constrained according to the exo-metabolomic profiles only, or increasing the cutoff for the generation of absent and present calls (*p* < 0.01), did not yield the same insights as presented herein (File S1, Table S18). Only recently Gene Inactivation Moderated by Metabolism, Metabolomics and Expression (GIM(3)E) became available, which enforces minimum turnover of detected metabolites based on intracellular metabolomics data as well as gene expression microarray data (Schmidt et al. 2013). In contrast to this approach, we emphasized our analysis on the relative differences in the exo-metabolomic data of two cell lines. GIM(3)E constitutes another integration method when the analysis should be emphasized on intracellular metabolomics data (Schmidt et al. 2013).

The metabolic differences predicted by the models are generally plausible. Cancers are known to be heterogeneous (Cairns et al. 2011), and the contribution of oxidative phosphorylation to cellular ATP production may vary (Zu and Guppy 2004). Moreover, leukemia cell lines have been shown to depend on glucose, glutamine, and fatty acids to varying extents to support proliferation. Such dependence may cause the cells to adapt their metabolism to the environmental conditions (Suganuma et al. 2010). In addition to identifying supporting data in the literature, we performed several analyses to validate the models and model predictions. Our expectations regarding the levels and ratios of metabolites relevant to energy and redox state were largely met (Fig. 4L). The more pronounced shift of the NADH/NAD+ ratio toward NADH in the CCRF-CEM cells was in agreement with the predicted Warburg phenotype (Fig. 4), and the higher lactate secretion in the CCRF-CEM cells (File S2, Fig. S2) implies an increase in NADH relative to NAD+ (Chiarugi et al. 2012; Nikiforov et al. 2011), again matching the known Warburg phenotype.

ROS production is enhanced in certain types of cancer (Droge 2002; Ha et al. 2000), and the generation of ROS is thought to contribute to mutagenesis, tumor promotion, and tumor progression (Dreher and Junod 1996; Ha et al. 2000). However, decreased mitochondrial glucose oxidation and a transition to aerobic glycolysis protect cells against ROS damage during biosynthesis and cell division (Brand and Hermfisse 1997). The higher ROS detoxification capability in Molt-4 cells, in combination with higher spermidine dismutase utilization by the Molt-4 model (Fig. 4), provided a consistent picture of the predicted respiratory phenotype (Fig. 4L).

Control of NADPH maintains the redox potential through GSH and protects against oxidative stress, yet changes in the NADPH ratio in response to oxidative damage are not well understood (Ogasawara et al. 2009). Under stress conditions, as assumed for Molt-4 cells, the NADPH/NADP+ ratio is expected to decrease because of the continuous reduction of GSSG (Fig. 4L), and this was confirmed in the Molt-4 cells (Fig. 4). The higher amounts of GSH found in Molt-4 cells in vitro may demonstrate an additional need for ROS scavengers because of a greater reliance on oxidative metabolism.

Cancer is related to metabolic reprogramming, which results from alterations of gene expression and the expression of specific isoforms or splice forms to support proliferation (Cortes-Cros et al. 2013; Marin-Hernandez et al. 2009). The gene expression differences detected between the two cell lines in the present study supported the existence of metabolic differences in these cell lines, particularly because key steps of the metabolic pathways central to cancer metabolism seemed to be differentially regulated (Table 1). The detailed analysis of the respective differences on the pathway fluxes exceeds the scope of this study, which was to demonstrate the potential of the integration of exo-metabolomic data into the network context.

We found discrepancies between differential gene regulation and the flux differences between the two models as well as the utilization AS gene-associated reaction. This is not surprising, since analysis of the detailed system is required to make any further assumptions on the impact that the differential regulation or splicing might have on the reaction flux, given that for many of the concerned enzymes isozymes exist, or only one of multiple subunits of a protein complex was concerned. Additionally, reaction fluxes are regulated by numerous post-translational factors, e.g., protein modification, inhibition through proteins or metabolites, alter reaction fluxes (Lenzen 2014), which are out of the scope of constraint-based steady-state modeling. Rather, the results of the presented approach demonstrate how the models can be used to generate informed hypothesis that can guide experimental work.

The combination of our tailored metabolic models and differential gene expression analysis seems well-suited to determine the potential drivers involved in metabolic differences between cells. Such information could be valuable for drug discovery, especially when more peripheral metabolic pathways are considered. Additionally, statistical comparisons of gene expression data with sampling-derived flux data could be useful in future studies (Mardinoglu et al. 2013).

A single-gene-deletion analysis revealed that PGDH was a lethal KO gene for the Molt-4 model only. Differences in PGDH protein levels correspond to the amount of glycolytic carbon diverted into glycine biosynthesis. Rapidly proliferating cells may use an alternative glycolytic pathway for ATP generation, which may provide an advantage in the case of extensive oxidative phosphorylation and proliferation (Locasale et al. 2011; Vander Heiden 2011; Vazquez et al. 2011). For breast cancer cell lines, variable dependency on the expression of PGDH has already been demonstrated (Locasale et al. 2011). This example of a unique KO gene demonstrates how in silico gene deletion in metabolomics-driven models can identify the metabolic pathways used by cancer cells. This approach can provide valuable information for drug discovery.

In conclusion, our contextualization method produced metabolic models that agreed in many ways with the validation data sets. The analyses described in this study have great potential to reveal the mechanisms of metabolic reprogramming, not only in cancer cells but also in other cells affected by diseases, and for drug discovery in general.

## Materials and methods

### Global model

The model we used (global model) was a subset of Recon 2 (Thiele et al. 2013), which is freely available (http://humanmetabolism.org/). Transport and exchange reactions for metabolites identified according to metabolite uptakes and secretions detected herein were already considered in the construction of Recon 2. The model captured 19 additional reactions (File S1, Table S10).

### Cell culture

MOLT-4 and CCRF-CEM cells were obtained from ATCC (CRL-1582 and CCL-119) and grown by standard methods in RPMI 1640, with 2 mM GlutaMax and 10 % FBS (Invitrogen; 61870-010, 10108-57), in a humidified incubator at 37 °C under 5 % CO_2_. At least 3 days before experiments were conducted, cells were introduced to serum-free medium (Advanced RPMI 1640, containing 2 mM GlutaMax; Invitrogen; 12633-012, 35050-038). The medium was refreshed the day before starting the experiment. For experiments, cells were centrifuged at 201×*g* for 5 min and resuspended in serum-free medium containing DMSO (0.67 %) at a cell concentration of 5 × 10^5^ cells/mL. The cell suspension was seeded in triplicate, with 1 or 2 mL applied to a 24-well or 12-well plate, respectively. At the indicated times, the cells were removed by centrifugation, and the spent medium was frozen at −80 °C. Cell number, size, and viability (Trypan blue exclusion) were determined by counting cells on a Countess automatic cell counter (Invitrogen).

### Analysis of the extracellular metabolome

Mass spectrometry analysis of the exo-metabolome was performed by Metabolon^®^, Inc. (Durham, NC, USA) using a standardized analytical platform. In total, 75 extracellular metabolites were detected in the initial data set for at least 1 of the 2 cell lines (Paglia et al. 2012a). Of these metabolites, 15 were not part of our global model and were discarded. Apart from being absent in our global model, an independent search in HMDB (Wishart et al. 2013) revealed no pathway information was available for most of these metabolites (File S1, Tables S2–S3). It should be noted that metabolites e.g., *N*-acetylisoleucine, *N*-acetylmethionine or pseudouridine, constitute protein and RNA degradation products, which were out of the scope of the metabolic network.

Thiamin (Vitamin B1) was part of the minimal medium of essential compounds supplied to both models. Riboflavin (Vitamin B2) and Trehalose were excluded since these compounds cannot be produced by human cells. Erythrose and fructose were also excluded. In contrast 46 metabolites that were part of the global model. The data set included two different time points, which allowed us to treat the increase/decrease of a metabolite signal between time points as evidence for uptake or secretion when the change was greater than 5 % from what was observed in the control (File S1, Tables S2–S3). We found 12 metabolites that were taken up by both cell lines and 10 metabolites that were commonly secreted by both cell lines over the course of the experiment. Additionally, Molt-4 cells took up three metabolites not taken up by CCRF-CEM cells, and secreted one metabolite not secreted by CCRF-CEM cells. Two of the three uniquely uptaken metabolites were essential amino acids: valine and methionine. However, it is unlikely that these metabolites were not taken up by the CCRF-CEM cells, and the CCRF-CEM model was allowed to take up this metabolite. Because of this adjustment, no quantitative constraints were applied for the sampling analysis either. CCRF-CEM cells had four unique uptaken and seven unique secreted metabolites (exchange not detected in Molt-4 cells).

### Network refinement based on exo-metabolic data

Despite its comprehensiveness, the human metabolic reconstruction is not complete with respect to extracellular metabolite transporters (Sahoo et al. 2014; Thiele et al. 2013). Accordingly, we identified metabolite transport systems from the literature for metabolites that were already part of the global model, but whose extracellular transport was not yet accounted for. Diffusion reactions were included whenever a respective transporter could not be identified. In total, 34 reactions [11 exchange reactions, 16 transport reactions and 7 demand reactions (File S1, Table S11)] were added to Recon 2 (Thiele et al. 2013), and 2 additional reactions were added to the global model (File S1, Table S10).

### Expression profiling

Molt-4 and CCRF-CEM cells were grown in advanced RPMI 1640 and 2 mM GlutaMax, and the cells were resuspended in medium containing DMSO (0.67 %) at a concentration of 5 × 10^5^ cells/mL. The cell suspension (2 mL) was seeded in 12-well plates in triplicate. After 48 h of growth, the cells were collected by centrifugation at 201×*g* for 5 min. Cell pellets were snap-frozen in liquid N2 and kept frozen until RNA extraction and analysis by Aros (Aarhus, Denmark).

### Analysis of transcriptomic data

We used the Affymetrix GeneChip Human Exon 1.0 ST Array to measure whole genome exon expression. We generated detection above background (DABG) calls using ROOT (version 22) and the XPS package for R (version 11.1), with Robust Multi-array Analysis summarization. Calls for data mapping were assigned based on *p* < 0.05 as the cutoff probability to distinguish presence versus absence for the 1,278 model genes (File S1, Table S12).

Differential gene expression and alternative splicing analyses were performed by using AltAnalyse software (v2.02beta) with default options on the raw data files (CEL files). The *Homo sapiens* Ensemble 65 database was used, probe set filtering was kept as DABG *p* < 0.05, and non-log expression < 70 was used for constitutive probe sets to determine gene expression levels. For the comparison, CCRF-CEM was the experimental group and Molt-4 was the baseline group. The set of DEGs between cell lines was identified based on a *p* < 0.05 FDR cutoff (File S1, Table S13A–B). Alternative splicing analysis was performed on core probe sets with a minimum alternative exon score of 2 and a maximum absolute gene expression change of 3 because alternative splicing is a less critical factor among highly DEGs (File S1, Table S14).

Gene expression data, complete lists of DABG *p*-values, DEGs and alternative splicing events have been deposited in the Gene Expression Omnibus (GEO) database (Accession number: GSE53123).

### Deriving cell-type-specific subnetworks

Transcriptomic data were mapped to the model in a manual fashion (COBRA function: deleteModelGenes). Specifically, reactions dependent on gene products that were called as “absent” were constrained to zero, such that fluxes through these reactions were disabled. Submodels were extracted based on the set of reactions carrying flux (network pruning) by running fastFVA (Gudmundsson and Thiele 2010) after mapping the metabolomic and transcriptomic data using the COBRA toolbox (Schellenberger et al. 2011).

### Cell weight

We calculated the cell dry weight based on the relative volume difference and comparison to human osteosarcoma (U2OS) cells. The cell dry weight of U2OS cells, ~60 pg (Mir et al. 2011), and cell volume, 4,000 µm^3^ (Beck et al. 2011), were derived from the literature. The cell volume of lymphocytes [243 µm^3^, the average volume of lymphoblasts from patients with ALL, (Chapman et al. 1981)] was derived from the literature. Cell dry weight was calculated accordingly: 4,000/243 = 16.46, and 60 pg/16.46 = 3.645 pg (3.645 × 1e^−12^ g).

### Definition of maximum uptake rate and minimum uptake rate

The maximum uptake rate was defined by the RPMI medium concentrations, and the minimum uptake was defined by mass spectrometry detection limits. Therefore, both medium concentration (mM) and detection limit (mM) were converted to flux values (mmol/gDW/h) by using a cell concentration of 2.17 × 1e^6^ (the concentration of viable CCRF-CEM cells after 48 h), an experimental duration of 48 h, and the calculated dry weight of 3.645 × 1e^−12^ g per cell: Flux = MetConc/(CellConc × CellWeight × T × 1,000). In the case of uptake, they were defined by the RPMI medium concentration (lower bound, lb) and the detection limit (upper bound, ub), and in the case of secretion, they were defined by the detection limit (lb) or left unconstrained (ub).

### Setting general and qualitative exo-metabolomic constraints during model building

Medium concentration to flux calculations were based on 3.645 × 1e^−12^ g cell weight, an initial cell concentration of 2.17 × 1e^6^, T = 48 h, and Flux = MetConc/(CellConc × CellWeight × T). We constrained the model by enforcing minimal flux through exchange reactions for secreted or uptaken metabolites in the correct directions (qualitative constraints). In the case of uptake, the upper bound of the corresponding exchange reaction was set to the flux equivalent of the minimal detection limit (Paglia et al. 2012a) using the same equation used for the concentrations in the medium. In the case of secretion, the lower bound of the exchange was set to be the minimum flux value based on the minimal detection limit (File S1, Table S15). The biomass reaction was constrained in a cell-line-specific manner. The experimental growth rate was 0.035 h^−1^ for CCRF-CEM and 0.032 h^−1^ for Molt-4 (File S1, Table S16). Vmax and Vmin were set to allow 20 % deviation from the experimental growth rate in each direction. Oxygen uptake was constrained to Vmin = −2.346 mmol/gDW/h (Thiele et al. 2005). All infinite fluxes were set to the maximum: −500/500 mmol/gDW/h. Alanine and glutamine are the breakdown products of GlutaMax in an external reaction. The model did not account for these reactions. However, the glutamine concentration was used to calculate the uptake flux of glutamine, which otherwise was not present in the medium. The increase of both compounds therefore did not necessarily reflect actual secretion by the cells, as it may have simply reflected the breakdown of GlutaMax, although additional secretion by the cells cannot be excluded. In the case of glutamine and alanine, the model exchanges remained unconstrained (qualitative and quantitative constraints) because the actual cell behavior could not be derived from the data, as it was overshadowed by accumulation resulting from the breakdown of GlutaMax (File S1, Tables S2–S3). Uptake of the conditionally essential amino acid cysteine (of which adequate amounts may not be produced) was enabled. Repeated profiling of the two cell lines supported the uptake of these amino acids (unpublished data). All other exchange reactions were constrained to zero, except those for basic ions, basic medium compounds and essential amino acids.

### Definition of quantitative constraints

The constraints on the exchange reactions defined during model building were the same in both condition-specific cell line models (Fig. 1D). For the analysis, we used the relative quantitative differences of commonly uptaken or secreted metabolites to further constrain the models (quantitative constraints). The model of the cell line that secreted more in the experiment was forced to secrete more by increasing the lower bound of the respective exchange reaction. The new lower bound was set to be proportionate to the difference in metabolite secretion in the experimental data (Fig. 1D, C, D). Accordingly, we decreased the lower bound of the model for the cell line that showed less uptake of the influx metabolites (Fig. 1D, A, B). For a list of the adjusted bounds, see the supplementary material (File S1, Table S17). To estimate the ratio for adjustment, we first calculated the fold change (FC) of each metabolite in the medium and in each cell line by comparing the zero and 48 h time points. Next, we compared the FC values to generate a slope (Slope = FCcelline/FCmedium) for each cell line. In the last step, we calculated the slope ratios (Slope Ratio = slopeCCRF-CEM/slopeMOLT4), which were used for the adjustments (Fig. 1D, colored x = Slope Ratio). Some metabolite exchanges were not adjusted, including those of phosphate and the essential amino acids histidine, l-cysteine, valine, methionine, alanine, and glutamine. The additional quantitative bounds were established to get a closer match to the phenotypes, so we refrained from adding constraints based on data, which was inconclusive.

Glutamine and alanine were the breakdown products of Glutamax, however instead of modeling the breakdown of Glutamax, we did not constrain the bounds for these compounds.

The ACHR sampler implemented in the COBRA toolbox (Schellenberger et al. 2011) was used with 10,000 generated warm-up points, nFiles = 100, pointsPerFile = 5,000, and stepsPerPoint = 2,500, and the cell-line models were used as inputs.

### Comparison of network utilization and DEGs/AS

The models shared a set of 1,907 reactions. We defined a reaction as differently utilized if the median value calculated from the sampling points differed by more than 10 %. The shared reaction set was divided into three groups: x (reactions with median difference >10 % and higher in CCRF-CEM cells) = 1,381, y (reactions with median difference >10 % and higher in Molt-4 cells) = 158, and z (reactions with median difference <10 % and reactions with opposite directionality in addition to loop reactions) = 368. Loop reactions were defined by FVA with the criteria minFlux = −500 and maxFlux = 500 (219 reactions in Molt-4, 220 reactions in CCRF-CEM).

### Enzyme assays

Molt-4 and CCRF-CEM cells were grown as described previously and harvested in the log growth phase. Cell number, size, and viability (Trypan blue exclusion) were determined by counting cells on a Countess automatic cell counter (Invitrogen). Cells were collected by centrifugation at 201×*g* for 5 min, washed once with PBS, and pelleted again by centrifugation. The cells were then resuspended in extraction buffer (0.1 M Tris, 2.5 mM EDTA, pH 7.75) to yield 1 × 10^5^ cells/µL. These cells were heated on a heat block set to 100 °C for 2 min, followed by cooling on ice. Following centrifugation at 20,000×*g*, the supernatant fraction (hereafter called the metabolite extract, ME) was removed and stored at −80 °C prior to biochemical assays. ATP content was measured in 100× diluted ME by using the CellTiter-Glo kit (Promega) and a Spectramax M3 microplate reader. NAD+ and NADH were measured in 5× diluted ME using the Amplite fluorometric NAD/NADH ratio assay kit (AAT Bioquest) according to the manufacturer’s instructions. NADP+ and NADPH were similarly measured by using the Amplite fluorometric NADP+/NADPH ratio assay kit (AAT Bioquest). Oxidized and reduced glutathione was measured similarly in 10× diluted ME by using the Amplite fluorometric GSH/GSSG ratio assay kit (AAT Bioquest). ROS was evaluated by using a modified ORAC assay based on a method described by Ganske and Dell (2006). Briefly, 25 µL of ME or 25 µL of the standard 6-hydroxy-2,5,7,8-tetra-methylchroman-2-carboxylic acid (Trolox, Sigma) was mixed with 150 µL of 10 nM fluorescein (Sigma) and 25 µL of 120 nM [2,2′-azobis(2-methylpropionamidine) dihydrochloride] (Sigma) in a transparent 96-well microplate (Brandt). Following 15 s of mechanical shaking, fluorescence (ex: 485 nm, em: 580 nm; 515 nm cutoff filter used for emission to improve signal) was monitored at 1-min intervals for 80 min at 37 °C. ORAC values were extrapolated from a Trolox standard curve by using Softmax Pro software and expressed as µmol of Trolox equivalent (μmol T.E.)/1 × 10^6^ cells. All biochemical assay data shown represent triplicate averages, n = 2.

All calculations were performed by using TomLab cplex linear solver and MATLAB.

## Electronic supplementary material

Below is the link to the electronic supplementary material.

File S1. Supplementary material 1 (XLSX 915 kb)

File S2. Supplementary material 2 (DOCX 448 kb)
