# Supplementary material for: Prediction of intracellular metabolic states from extracellular metabolomic data
Source: Metabolomics. 2014 Aug 14;11(3):603–19. doi: 10.1007/s11306-014-0721-3 (PMC4419158; doi:10.1007/s11306-014-0721-3)
Supplement: Supplementary file 2 — File S2. Supplementary material 2 (DOCX 448 kb) [file 11306_2014_721_MOESM2_ESM.docx]

**Prediction of intracellular metabolic states from extracellular metabolomic data**

Maike K. Aurich^1,3^, Giuseppe Paglia^1^, Óttar Rolfsson^1^, Sigrún Hrafnsdóttir^1^, Manuela Magnúsdóttir^1^, Magdalena M. Stefaniak^2^, Bernhard Ø. Palsson^1,4^, Ronan M.T. Fleming^1,3^, Ines Thiele^1,3,*^

^1^ Center for Systems Biology, University of Iceland, Reykjavik,

Iceland

^2^ School of Health Sciences, Faculty of Food Science and Nutrition, University of Iceland, Reykjavik, Iceland

^3^ Luxembourg Centre for Systems Biomedicine, University of Luxembourg, Campus Belval, Esch-sur-Alzette, Luxembourg.

^4^ Department of Bioengineering, University of California San Diego, La Jolla, California, United States of America.

**Supplementary material**

**Contents**

1. **Supplementary Figures**

**Figure S1: Growth and apoptosis of MOLT4 and CCRF-CEM cells.**

**Figure S2:** **Sampling reveals different utilization of glycolysis by the two models.**

**2) Supplementary Results**

**Supplementary Figures:**


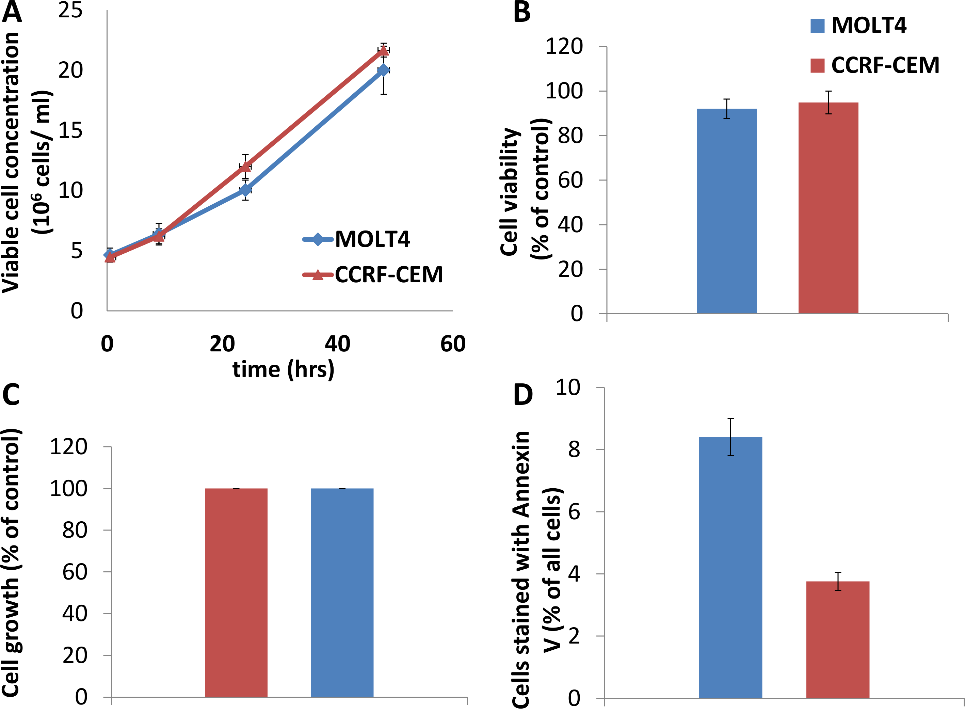


**Figure S1: Growth and apoptosis of MOLT4 and CCRF-CEM cells.** Cells were resuspended in RPMI advanced containing DMSO (0.67%) and cultured at 37°C and 5% CO2 in 24 well plates. **A** Cells were counted using an automatic cell counter. The graphs show viable cells (excluding Trypan blue). Data shown is the average and standard deviation of biological triplicates. **B** Inhibition of growth by DMSO (0.67%) after 48 hrs. Data shown is the average and standard deviation of 7 (CCRF-CEM) and 6 (Molt-4) independent experiments. **C** Cell viability is shown as the fraction of cells excluding Trypan blue in each sample. Data shown is the average and standard deviation of 7 (CCRF-CEM) and 6 (Molt-4) independent experiments. **D** Apoptosis was measure after 48 hrs using Annexin V binding and flow cytometry. Data shown are all apoptotic cells, including cells undergoing necrosis (stained with Annexin V-PE and 7-AAD). Data shown is the average and standard deviation of three independent experiments.


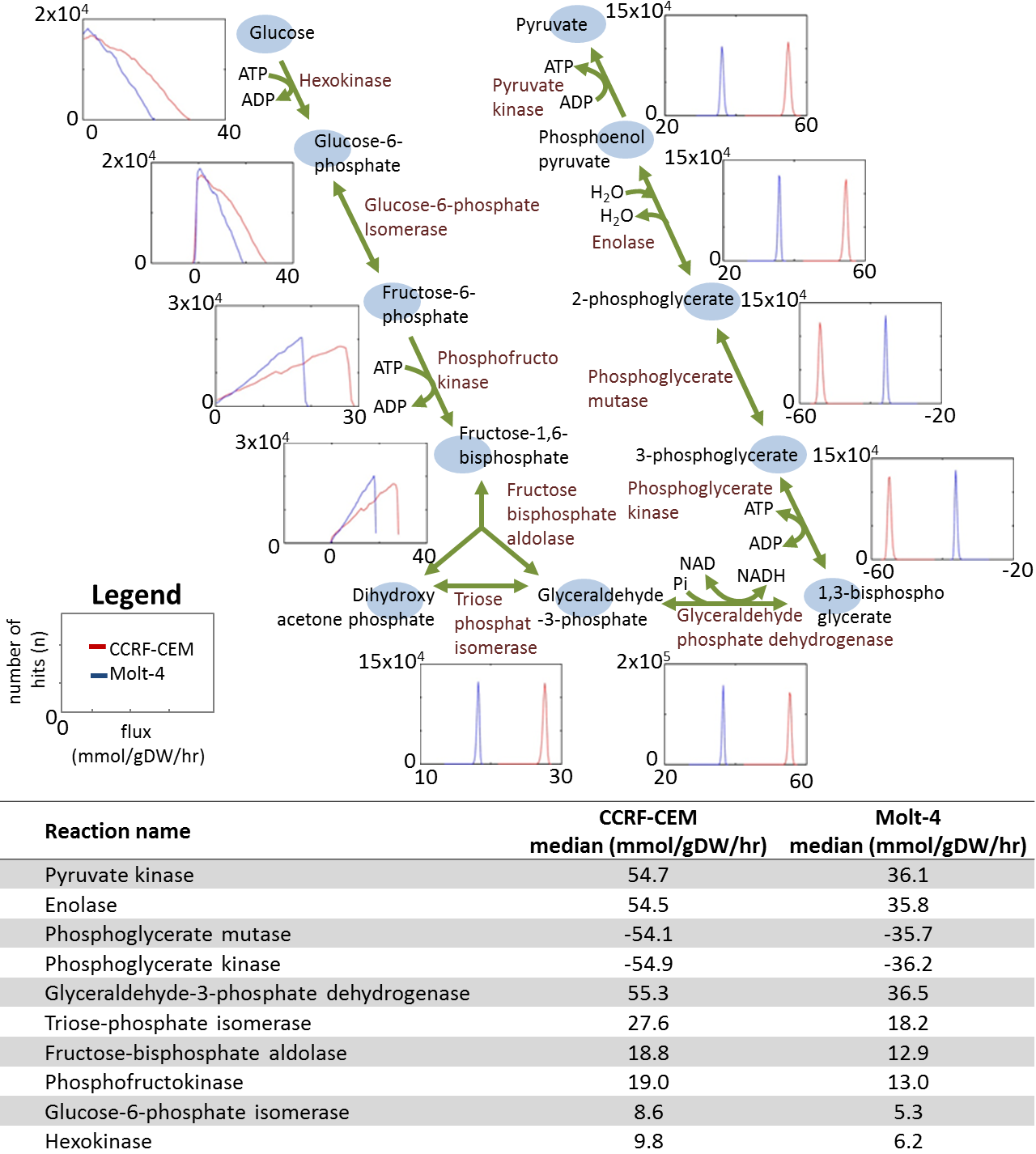


**Figure S2: Sampling reveals different utilization of glycolysis by the two models.** Histograms of sampling points were different between the CCRF-CEM model (red) and the Molt-4 model (blue) for 10 glycolysis reactions. Negative values in the histograms and the table describe reaction fluxes in the reverse direction of reversible reactions. The table provides the median values of the sampling results.

**Supplementary Results**

**Models cover equal amounts of differentially expressed and alternatively spliced genes**

The GeneChip Human Exon 1.0 ST Array had been used to measure gene expression and exon variation in Molt-4 and CCRF-CEM cells. We derived sets of differentially expressed (DEGs) and alternatively spliced (AS) genes by comparing gene expression between the two cell lines.

The analysis yielded 57 Recon 1 genes with significantly more expressed in CCRF-CEM compared to Molt-4 cells (upregulated), and 16 genes with significantly lower expression in CCRF-CEM compared to Molt-4 cells (downregulated). To validate the models, we investigated how many of the genes remained part of the condition specific models, after the integration of the metabolomic data. CCRF-CEM and the Molt-4 specific model covered the same subset of DEGs, both of 49 (66 transcripts) upregulated genes and 12 (13 transcripts) downregulated genes (missing downregulated genes Entrez Gene ID: 19, 875, 23657, 2944; missing upregulated genes Entrez Gene ID: 64131, 64772, 2581, 256435, 6799, 4697, 9951, 7263), and also the same amount of reactions associated with these DEGs, which was 144 reactions for upregulated genes, and 15 reactions associated with downregulated genes (Figure 1C).

We identified 90 AS genes in the set of Recon 1 genes, and all AS genes remained in the condition specific models. CCRF-CEM and Molt-4 models both covered an equal set of 211 AS gene associated reactions (Figure 1C).

The gene expression data contributed only minor to the differentiation of the models. Both models included the same amount of DEGs and AS genes, as well as the same amount of reactions associated with these gene sets.
